# Laboratory protocol for the digital multiplexed gene expression analysis of nasopharyngeal swab samples using the NanoString nCounter system

**DOI:** 10.12688/f1000research.103533.1

**Published:** 2022-02-02

**Authors:** Marilina García Aranda, Inmaculada López-Rodríguez, Susana García-Gutiérrez, Maria Padilla-Ruiz, Vanessa de Luque, Maria Luisa Hortas, Tatiana Diaz, Martina Álvarez, Isabel Barragan-Mallofret, Maximino Redondo, Research Network on Health Services in Chronic Diseases (Red de Investigación en Servicios Sanitarios y Enfermedades Crónicas, REDISSEC)

**Affiliations:** 1 Research and Innovation Unit, Hospital Costa del Sol, Marbella, 26903, Spain; 2 Surgery, Biochemistry and Immunology Department, University of Malaga, Malaga, 29071, Spain; 3 Translational Research in Cancer and Other Prevalent Chronic Diseases Group, Instituto de Investigación Biomédica de Málaga (IBIMA), Malaga, Spain; 4 Microbiology Unit (Clinical Analysis Laboratory), Hospital Costa del Sol, Marbella, 26903, Spain; 5 Kronikgune, Research Unit, Hospital Galdakao-Usansolo, Galdakao, Spain; 6 UGC Oncología Médica. Hospitales Universitarios Regional y Virgen de la Victoria, Instituto de Investigación Biomédica de Málaga (IBIMA), University of Malaga, Malaga, Spain; 7 Clinical Laboratories Area, Hospital Costa del Sol, Marbella, 26903, Spain; 8 Andalusian Public Health System Biobank, Instituto de Investigación Biomédica de Málaga (IBIMA), Malaga, Spain; 9 Translational Research in Immunotherapy for Cancer Group, Medical Oncology Intercenter Unit, Regional and Virgen de la Victoria University Hospitals, Instituto de Investigación Biomédica de Málaga (IBIMA), Malaga, 29010, Spain; 10 Group of Pharmacoepigenetics, Department of Physiology and Pharmacology, Karolinska Institutet, Stockholm, Sweden

**Keywords:** respiratory infection, nasopharyngeal swab, gene expression, Immunology, Digital RNA quantification

## Abstract

This paper describes a laboratory protocol to perform the NanoString nCounter Gene Expression Assay from nasopharyngeal swab samples.

It is urgently necessary to identify factors related to severe symptoms of respiratory infectious diseases, such as COVID-19, in order to assess the possibility of establishing preventive or preliminary therapeutic measures and to plan the services to be provided on hospital admission. At present, the samples recommended for microbiological diagnosis are those taken from the upper and/or the lower respiratory tract.

The NanoString nCounter Gene Expression Assay is a method based on the direct digital detection of mRNA molecules by means of target-specific, colour-coded probe pairs, without the need for mRNA conversion to cDNA by reverse transcription or the amplification of the resulting cDNA by PCR. This platform includes advanced analysis tools that reduce the need for bioinformatics support and also offers reliable sensitivity, reproducibility, technical robustness and utility for clinical application, even in RNA samples of low RNA quality or concentration, such as paraffin-embedded samples. Although the protocols for the analysis of blood or formalin-fixed paraffin-embedded samples are provided by the manufacturer, no corresponding protocol for the analysis of nasopharyngeal swab samples has yet been established. Therefore, the approach we describe could be adopted to determine the expression of target genes in samples obtained from nasopharyngeal swabs using the nCOUNTER technology.

## Introduction

### Nasopharyngeal swabs as valuable biospecimens

Nasopharyngeal swabs are essential for the accurate diagnosis of respiratory infectious diseases such as coronavirus disease 2019 (COVID-19), which is caused by the severe acute respiratory syndrome coronavirus 2 (SARS-CoV-2). The samples currently recommended for the microbiological diagnosis of respiratory infectious diseases are those obtained from the upper respiratory tract (nasopharynx and oropharynx) and/or the lower respiratory tract, such as sputum, endotracheal aspirates, bronchoalveolar lavages or bronchial aspirates, especially in patients with severe respiratory disease.

Although specimen collection can be an uncomfortable procedure for the patient it provides a valuable mixture containing biological material both from the infectious agent and from the patient. The high viral load obtained, ^1^ the simplicity of the procedure involved and the ready availability of this type of sample in laboratories performing routine microbiological analyses make surplus biospecimens a valuable source of biologic material for conducting molecular or genetic studies of the infectious agent and the host.

### Digital multiplexed RNA quantification

In recent years, the study of selected genes by real-time PCR or genome-wide gene expression microarray analysis has been used in genetic research to detect associations between specific gene expression profiles and particular diseases. Within these technologies, the nCOUNTER ^®^ platform ( NanoString Technologies, Seattle, WA) delivers direct and multiplexed measurement of gene expression, providing digital readouts of the relative abundance of mRNA transcripts simultaneously ^2^ in a single assay, without the need for cDNA conversion or amplification of target RNA. This platform, which offers reliable sensitivity, reproducibility, technical robustness and utility for clinical application, ^3^ is also capable of analysing RNA samples of poor quality such as fragmented RNA (35 to 50-base target-specific sequences) or cell lysates with total RNA concentrations as low as 100 ng, ^4^ as is foreseeably the case with samples from nasopharyngeal swabs.

The nCOUNTER Human Immunology V2 CSO panel, which facilitates the study of 594 genes, including the major classes of cytokines, chemokine ligands, interferons and their receptors, the TNF-receptor superfamily, the KIR family genes and genes involved with the anti-fungal immune response, is recommended for the study of the immune response to infectious disease in samples with fragmented RNA or low RNA inputs. ^5^ This panel can also be combined with an additional panel of 55 genes related to the human inflammatory response. Although the protocols for the study of blood or formalin-fixed paraffin-embedded samples are well known and provided by the manufacturer, no protocol for the analysis of nasopharyngeal swab samples has yet been established.

## Protocol

### Patients

Our study will include 250 patients admitted to the Hospital Costa del Sol (Marbella, Spain) with severe COVID-19 and positive PCR results for SARS-CoV-2. To participate in the study, all patients will receive a patient information sheet and will be asked to sign the corresponding informed consent form.

### Obtention of biologic samples

The procedure for the routine determination of SARS-CoV-2 by PCR includes taking a nasopharyngeal sample with the sterile, fine, flexible swab that is included in the specific respiratory sampling kit for viruses. According to the protocol stipulated by the Spanish Ministry of Health, ^1^ during sampling, the swab must be introduced through the nostril, parallel to the palate, to a depth equal to the distance from the nostrils to the outer opening of the ear. The swab should be maintained inside the nostril for five seconds to allow absorption of the secretions and should then be withdrawn slowly while making 180° rotations. After taking the sample, the swab should immediately be placed in a sterile tube with 2-3 ml of viral transport medium and kept refrigerated at +4°C until it is analysed at the molecular biology and microbiology laboratory.

Various kits are currently marketed for the collection, transport, and maintenance of clinical samples until the laboratory analysis is performed, some of which include transport media with an inactivator. In the subsequent analysis of the results, it must be considered whether the use of one or other means of transport might affect the final result.

We have performed a local validation study of a subset of samples to confirm that the viral transport media brands used in our laboratory (δ-Swab ^TM^; UTM ^TM^-Viral Transport Media; Viroclinics Biosciences, Mwe Viral Transport Media, Vircell Transport Media) do not compromise the results obtained.

### Heat inactivation protocol

To safely handle biological samples, they must first be inactivated. With samples obtained from nasopharyngeal swabs for molecular analysis, this is usually accomplished by the addition of a chemical quencher or by heat treatment.

Given the low concentration of genetic material in nasopharyngeal swabs, together with the high presence of biologic contaminants in upper respiratory airways, we recommend heat-treatment inactivation. Various techniques have been described to perform this task without affecting the integrity of the RNA, including inactivation at 56°C for 30 minutes, at 65°C for 15 minutes, at 95°C for 5 minutes or at 98 °C for 2 minutes. ^6^^,^^7^

Before processing the samples, it is necessary to ensure that thermal inactivation does not impair RNA integrity, which can be performed by comparing the performance of RT-PCR analysis for SARS-CoV-2 after treating a set of samples to each of the heat inactivation protocols. In our study, we performed a local validation of a subset of samples with a volume of 400 μl, which confirmed that RT-PCR sensitivity is not compromised by heat inactivation at 98°C for 2 minutes.

### Nucleic acid extraction protocol

We performed the local validation of a subset of samples to assess the performance of both the manual and the automated procedures considered. In the first case, the extraction was performed using the RNEasy kit ( Qiagen, Hilden, Germany), according to the manufacturer's instructions, using an initial sample volume of 500 μl and a final volume of 10 μl. For automated extraction, we tested the Biorobot EZ1, also obtained from Qiagen, with an initial sample volume of 200 μl and a final volume of 60 μl. Finally, RNA extraction was performed in the MagCore robot (Magcore Lamination India Pvt. Ltd), with an initial sample volume of 200 μl or 400 μl and a final volume of 40 μl.

NanoString input recommendations stipulate a total RNA concentration range of 100-125 ng and specific purity ratios of absorbance measured by spectrophotometry at 260, 280 and 230nm. Since aromatic proteins have a strong UV absorbance at 280 nm, the A260/280 ratio is generally used to assess protein contamination in a nucleic acid sample. A260/280 ratios under 1.7 indicate the presence of contaminants that can affect the result, being the A260/280 ratio of ~2.0 as the generally accepted as “pure” for RNA. In such a manner, the A260/230 ratio is used to reveal the presence of organic contaminants such as phenol or TRIzol. Generally acceptable A260/230 ratios are those in the range of 2.0-2.2.

In our local validation study, we obtained varying results for RNA concentration and purity ( Table 1). As can be seen, the eluates obtained by manual extraction had neither the concentration nor the purity required by the equipment. Automated extraction with the Qiagen EZ1 kit also produced aliquots of insufficient purity, which could invalidate the analysis results in the nCounter. Finally, using the MagCore equipment, for the same final eluate volume of 40 μl, initial sample volumes of 200 μl and 400 μl were tested, with the latter obtaining the best results.

**Table 1.** Performance of four different ARN extraction procedures.

Procedure	Kit name	Sample input	RNA Concentration (ng/μl)	A260/280	A260/230
Manual	RNEasy (Qiagen ^®^)	Initial sample volume, 500 μl. Final eluate volume, 10 μl.	7.03 (SD 9.53)	1.25 (SD 0.39)	0.22 (SD 0.25)
Automated	Kit EZ1 Virus Mini Kit v2.0 de Qiagen ^®^	Initial volume 400 μl. Final volume 60 μl.	259.34 (SD 66.49)	3.20 (SD0.26)	0.84 (SD 0.17)
	Kit MagCore ^®^ Viral Nucleic Acid Extraction Kit Cartridge Code 203 (HF16, Compact)	Initial sample volume, 200 μl. Final eluate volume, 40 μl.	28.39 (SD13.47)	2.14 (SD 0.36)	1.12 (SD 0.51)
	Kit MagCore ^®^ Viral Nucleic Acid Extraction Kit Cartridge Code 203 (HF16, Compact)	Initial sample volume, 400 μl. Final eluate volume, 40 μl.	43.60 (SD 36.1)	1.90 (SD 0.13)	1.53 (SD 0.40)

The A260/280 ratio is used to assess protein contamination. For pure RNA, the recommended A260/280 ratio should be ~2.0. The A260/230 ratio is used to assess the presence of organic contaminants. A260/230 ratios under 1.8 indicate the presence of contaminants. For RNA, the recommended A260/230 ratio should also be ~2.0.

SD (Standard deviation).

### Gene expression

We performed a local validation study in a subset of purified RNA aliquots from heat-inactivated nasopharyngeal swabs, in order to evaluate the performance of the NanoString nCounter platform in analysing the expression of our target genes, and obtained satisfactory results.

We followed the manufacturer’s instructions, using 100 ng of total RNA. In summary, the protocol consists of the following steps:

1. Preheat the thermocycler to 65°C. Thaw the kit reagents and samples for 30 minutes.
2. Add 70 μl of hybridisation buffer + 28 μl of Reporter Plus to the Reporter Codeset tube. Mix gently.
3. Aliquot 10 μl of Master Mix to each well of the 12-tube strip.
4. Add 5 μl of the samples to each corresponding well (RNA concentration should be 100 μg/μl. Dilute out-of-range samples with RNAse-free water).
5. Spin the Capture ProbeSet and Capture Plus tubes.
6. Add 14 μl of Capture Plus to the Capture ProbeSet tube to create the Master Capture. Mix gently and spin the Master Mix.
7. Add 3 μl of Master Capture to each well of the 12-tube strip.
8. Cover the tube strip with a corresponding plug strip. Mix gently and spin slowly so that the entire volume drops to the bottom of the well, leaving no bubbles.
9. Use the thermocycler to perform hybridisation with the reporter and capture probes that carry the fluorescent signals, for 16-24 hours.
10. Combine pairs of probes specific for the selected genes with a series of internal controls to form a molecular barcoding, or CodeSet, allowing downstream digital detection.
11. Remove excess probes, align the probe/target complexes, immobilise them in the nCounter Cartridge, and then insert them into the nCounter Digital Analyser for data collection.

### Data analysis

The differential expression analysis data model preferentially applies the optimal statistical method per gene given the following variable distribution: 1) Mixture negative binomial model, 2) Simplified negative binomial model, 3) Log-linear model, in that order. FDR p-value adjustment will be performed according to the Benjamini-Hochberg method. All samples will be normalised using the geometric mean of the housekeeping genes.

## Conclusions

COVID-19 is a major global health problem, making it necessary to develop tools to optimise healthcare and facilitate personalised treatment. From a clinical perspective, the identification of gene transcripts related to the poor prognosis of patients hospitalised with SARS-CoV-2 has undeniable practical value. This complementary information would be straightforward to design multiplexed panels and prediction tools that can be incorporated into computers used in daily practice, helping clinicians predict and identify possible outcomes and facilitating decision-making in this respect.

Our study may also provide useful information to help establish the protocols of other studies based on the analysis of nasopharyngeal swab samples using the NanoString nCOUNTER platform.

## Data availability

Normalisation, differential expression analysis and pathway analysis can be performed with Nanostring nCounter nSolver™ 4.0 (RRID:SCR_003420), using the Nanostring Advanced Analysis Module 2.0 plugin and following the Nanostring Gene Expression Data Analysis Guidelines. Advanced Analysis Module 2.0 software uses open-source R programs for quality control, normalisation, differential data analysis, pathway scoring and gene-set enrichment analysis.

## Author contributions

MGA, SGR, IBM, MR conceptualization of the study. SGR, MGA, MR funding acquisition. MGA, ILR, TD, VDL, IBM, MA, MLH investigation and methodology. MPR, IBM, MGA, MA contributed to data analysis. MGA, IBM, MR supervised the study and revised the manuscript. All authors read and approved the final manuscript.

## Ethics

Our Institutional Review Board (CEI Costa del Sol exp.003_JUL20_PI-IMMU-COVID19) approved this study in July 2020. All patients will be informed of the study and invited to participate. All participation will be subject to the provision of informed written consent.
