# Laboratory protocol for the digital multiplexed gene expression analysis of nasopharyngeal swab samples using the NanoString nCounter system

**DOI:** 10.12688/f1000research.103533.2

**Published:** 2022-09-30

**Authors:** Marilina García Aranda, Inmaculada López-Rodríguez, Susana García-Gutiérrez, Maria Padilla-Ruiz, Vanessa de Luque, Maria Luisa Hortas, Tatiana Diaz, Martina Álvarez, Isabel Barragan-Mallofret, Maximino Redondo, Research Network on Health Services in Chronic Diseases (Red de Investigación en Servicios Sanitarios y Enfermedades Crónicas, REDISSEC)

**Affiliations:** 1 Research and Innovation Unit, Hospital Costa del Sol, Marbella, 26903, Spain; 2 Surgery, Biochemistry and Immunology Department, University of Malaga, Malaga, 29071, Spain; 3 Translational Research in Cancer and Other Prevalent Chronic Diseases Group, Instituto de Investigación Biomédica de Málaga y Plataforma en Nanomedicina-IBIMA Plataforma Bionand. C/ Marqués de Beccaria nº3, Malaga, 29010, Spain; 4 Microbiology Unit (Clinical Analysis Laboratory), Hospital Costa del Sol, Marbella, 26903, Spain; 5 Kronikgune, Research Unit, Hospital Galdakao-Usansolo, Galdakao, Spain; 6 UGC Oncología Médica. Hospitales Universitarios Regional y Virgen de la Victoria, Instituto de Investigación Biomédica de Málaga (IBIMA), University of Malaga, Malaga, Spain; 7 Medical Oncology Service (Group of Translational Research in Cancer Immunotherapy), Regional and Virgen de la Victoria University Hospitals, Instituto de Investigación Biomédica de Málaga y Plataforma en Nanomedicina–IBIMA Plataforma Bionand, C/ Marqués de Beccaría n°3, Malaga, 29010, Spain; 8 Clinical Laboratories Area, Hospital Costa del Sol, Marbella, 26903, Spain; 9 Andalusian Public Health System Biobank, Instituto de Investigación Biomédica de Málaga (IBIMA), Malaga, Spain; 10 Translational Research in Immunotherapy for Cancer Group, Medical Oncology Intercenter Unit, Regional and Virgen de la Victoria University Hospitals, Instituto de Investigación Biomédica de Málaga (IBIMA), Malaga, 29010, Spain; 11 Group of Pharmacoepigenetics, Department of Physiology and Pharmacology, Karolinska Institutet, Stockholm, Sweden

**Keywords:** respiratory infection, nasopharyngeal swab, gene expression, Immunology, Digital RNA quantification

## Abstract

This paper describes a laboratory protocol to perform the NanoString nCounter Gene Expression Assay from nasopharyngeal swab samples.

It is urgently necessary to identify factors related to severe symptoms of respiratory infectious diseases, such as COVID-19, in order to assess the possibility of establishing preventive or preliminary therapeutic measures and to plan the services to be provided on hospital admission. At present, the samples recommended for microbiological diagnosis are those taken from the upper and/or the lower respiratory tract.

The NanoString nCounter Gene Expression Assay is a method based on the direct digital detection of mRNA molecules by means of target-specific, colour-coded probe pairs, without the need for mRNA conversion to cDNA by reverse transcription or the amplification of the resulting cDNA by PCR. This platform includes advanced analysis tools that reduce the need for bioinformatics support and also offers reliable sensitivity, reproducibility, technical robustness and utility for clinical application, even in RNA samples of low RNA quality or concentration, such as paraffin-embedded samples. Although the protocols for the analysis of blood or formalin-fixed paraffin-embedded samples are provided by the manufacturer, no corresponding protocol for the analysis of nasopharyngeal swab samples has yet been established. Therefore, the approach we describe could be adopted to determine the expression of target genes in samples obtained from nasopharyngeal swabs using the nCOUNTER technology.

## Introduction

### Nasopharyngeal swabs as valuable biospecimens

The samples currently recommended for the microbiological diagnosis of respiratory infectious diseases are those obtained from the upper respiratory tract (nasopharynx and oropharynx) and/or the lower respiratory tract, such as sputum, endotracheal aspirates, bronchoalveolar lavages or bronchial aspirates, especially in patients with severe respiratory disease.

Nasopharyngeal swabs provide a valuable mixture containing biological material both from the infectious agent and from the patient. The high viral load obtained, ^1^ the simplicity of the procedure involved and the ready availability of this type of sample in laboratories performing routine microbiological analyses make surplus biospecimens a valuable source of biologic material for conducting molecular or genetic studies of the infectious agent and the host.

### Digital multiplexed RNA quantification

In recent years, the study of selected genes by real-time PCR or genome-wide gene expression microarray analysis has been used in genetic research to detect associations between specific gene expression profiles and particular diseases. Within these technologies, the nCOUNTER ^®^ platform ( NanoString Technologies, Seattle, WA) delivers direct and multiplexed measurement of gene expression, providing digital readouts of the relative abundance of mRNA transcripts simultaneously ^2^ in a single assay, without the need for cDNA conversion or amplification of target RNA. This platform, which offers reliable sensitivity, reproducibility, technical robustness and utility for clinical application, ^3^ is also capable of analyzing RNA samples of poor quality such as fragmented RNA (35 to 50-base target-specific sequences) or cell lysates with RNA concentrations as low as 100 ng, ^4^ as is foreseeable the case with samples from nasopharyngeal swabs.

The nCOUNTER Human Immunology V2 CSO panel, which facilitates the study of 594 genes, including the major classes of cytokines, chemokine ligands, interferons and their receptors, the TNF-receptor superfamily, the KIR family genes and genes involved with the anti-fungal immune response, is recommended for the study of the immune response to infectious disease in samples with fragmented RNA or low RNA inputs. ^5^ This panel can also be combined with an additional panel of 55 genes related to the human inflammatory response. Although the protocols for the study of blood or formalin-fixed paraffin-embedded samples are well known and provided by the manufacturer, no protocol for the analysis of nasopharyngeal swab samples has yet been established.

## Protocol

### Patients

Our study included 250 patients admitted to the Hospital Costa del Sol (Marbella, Spain) with severe COVID-19 and positive PCR results for SARS-CoV-2. To participate in the study, all patients received a patient information sheet and were asked to sign the corresponding informed consent form.

### Obtention of biologic samples

The procedure for the routine determination of SARS-CoV-2 by PCR includes taking a nasopharyngeal sample with the sterile, fine, flexible swab that is included in the specific respiratory sampling kit for viruses. According to the protocol stipulated by the Spanish Ministry of Health, ^1^ during sampling, the swab must be introduced through the nostril, parallel to the palate, to a depth equal to the distance from the nostrils to the outer opening of the ear. The swab should be maintained inside the nostril for five seconds to allow absorption of the secretions and should then be withdrawn slowly while making 180° rotations. After taking the sample, the swab should immediately be placed in a sterile tube with 2-3 ml of viral transport medium and kept refrigerated at +4°C until it is analyzed at the molecular biology and microbiology laboratory. In case of delay in processing, samples may also be frozen at -80°C until further analysis.

Various kits are currently marketed for the collection, transport, and maintenance of clinical samples until the laboratory analysis is performed, some of which include transport media with an inactivator. In the subsequent analysis of the results, we also considered whether the use of one or other viral transport mediums affected the final result.

### Heat inactivation protocol

To safely handle biological samples, they must first be inactivated. With samples obtained from nasopharyngeal swabs for molecular analysis, this is usually accomplished by the addition of a chemical quencher or by heat treatment.

Given the low concentration of genetic material in nasopharyngeal swabs, together with the high presence of biologic contaminants in upper respiratory airways, we recommend heat-treatment inactivation. Various techniques have been described to perform this task without affecting the integrity of the RNA, including inactivation at 56°C for 30 minutes, at 65°C for 15 minutes, at 95°C for 5 minutes or at 98°C for 2 minutes. ^6^ ^,^ ^7^

Before processing the samples, we ensured that thermal inactivation did not impair RNA integrity, by comparing the performance of RT-PCR analysis for SARS-CoV-2 after treating a set of samples to each of the heat inactivation protocols. In our experiment, all samples were inactivated at 98°C for 2 minutes as previously described.

### Nucleic acid extraction protocol

Following viral inactivation, RNA was immediately extracted. Given the novelty of the protocol, we performed a local validation of a subset of samples to assess the performance of both the automated and manual RNA extraction procedures.

For automated extraction, we tested the performance of the Biorobot EZ1 (Qiagen, Hilden, Germany), with an initial sample volume of 200 μl and a final eluate volume of 60 μl; and the MagCore robot (Magcore Lamination India PvT. Ltd), with initial sample volumes of 200 μl or 400 μl and a final eluate volume of 40 μl. Although these kits are not the most optimal methods to allow for the detection of host genes, our preliminary results demonstrate their validity to study host gene expression.

With respect to the manual RNA extraction procedure, and due to supply shortages during the COVID-19 pandemic, we were not able to use the recommended QIAMp MinElute Virus extraction kit (Qiagen). Instead, and according to previously published papers in this field, we tested the RNEasy Mini Kit (Qiagen), which is one of the most common commercial kits and the Gold Standard for RNA extraction, as an alternative strategy for viral detection in sputum ^8^ or nasopharyngeal samples. ^9^ We processed the samples according to manufacturer’s instructions, using an initial sample volume of 500 μl and a final volume of 10 μl.

Given its affordability, rapidity an ease of use, we firstly carried out a general assessment of eluates sample quality and concentration with the NanoDrop ^TM^2000 spectrophotometer (Themo Fisher Scientific, Waltham, Massachusetts). NanoString input recommendations stipulate a total RNA concentration range of 100-130 ng and specific ratios of absorbance at 260 nm and 280 nm (A260/280) measured with NanoDrop within the range 1.80-2 nm. Since aromatic proteins have a strong UV absorbance at 280 nm, the A260/280 ratio is generally used to assess protein contamination in a nucleic acid sample. A260/280 ratios under 1.7 usually indicate the presence of contaminants that can affect the result, being the A260/280 ratio of ~2.0 as the generally accepted as “pure” for RNA. In such a manner, the A260/230 ratio is normally used to reveal the presence of organic contaminants such as phenol or TRIzol. Generally acceptable A260/230 ratios are those in the range of 1.8-2.2.

In our local validation study, we obtained varying results for RNA concentration and absorbance ratios ( Table 1) for each of the studied procedures. As can be seen, eluted samples obtained by manual extraction had neither the concentration nor the purity required by the equipment. Automated extraction with the Qiagen EZ1 kit also produced aliquots of insufficient purity, which could invalidate the analysis results in the nCounter. Finally, using the MagCore equipment, for the same final elution volume of 40 μl, initial sample volumes of 200 μl and 400 μl were tested, with the latter obtaining the best results. The success rate of extractions performed with the MagCore robot was 86%.

**Table 1.** Performance of four different ARN extraction procedures measured with NanoDrop 2000 spectrophotometer.

Procedure	Kit name	N	Sample input	RNA Concentration (ng/μl)	A260/280	A260/230
Manual	RNEasy (Qiagen ^®^)	12	Initial sample volume, 500 μl. Final eluate volume, 10 μl.	7.03 (SD 9.53)	1.25 (SD 0.39)	0.22 (SD 0.25)
Automated	Kit EZ1 Virus Mini Kit v2.0 de Qiagen ^®^	14	Initial volume 400 μl. Final volume 60 μl.	259.34 (SD 66.49)	3.20 (SD 0.26)	0.84 (SD 0.17)
	Kit MagCore ^®^ Viral Nucleic Acid Extraction Kit Cartridge Code 203 (HF16, Compact)	16	Initial sample volume, 200 μl. Final eluate volume, 40 μl.	37.30 (SD 9.18)	2.08 (SD 0.42)	1.12 (SD 0.38)
	Kit MagCore ^®^ Viral Nucleic Acid Extraction Kit Cartridge Code 203 (HF16, Compact)	16	Initial sample volume, 400 μl. Final eluate volume, 40 μl.	49.17 (SD 26.54)	1.87 (SD 0.11)	1.40 (SD 0.29)

SD: Standard deviation.

Once we decided to use the MagCore extraction method, we performed a local validation study to assess RNA concentration and purity measured with NanoDrop ^TM^2000 spectrophotometer as well as RNA concentration and integrity measured with the Agilent 2100 System Bioanalyzer (Agilent Scientific Instruments Inc., Santa Clara, California) of a subset of 131 purified RNA aliquots from heat-inactivated nasopharyngeal swabs (2 minutes at 98°C) processed with the MagCore robot (initial sample volume of 400 μl and a final eluate volume of 40 μl), considering the different viral transport media brands in which patient samples were preserved (Deltaswab ViCUM ^®^; Deltaswab Virus ^®^; Sigma Virocult ^®^ Mwe; Vircell Transport Medium; Copan UTM ^®^-RT) ( Table 2).

**Table 2.** Results of RNA concentration and absorbance ratios measured with NanoDrop 2000, RNA integrity and concentration measured with Agilent Bioanalyzer and nCounter success rate and performance for each of the tested viral transport media brands.

Transport media	N	NanoDrop 2000			Agilent Bioanalyzer		nCounter NanoString	
		Concentration (ng/μl)	A260/280	A260/230	Concentration (pg/μl)	DV200	Panel validity	Panel Performance
Copan UTM-RT	37	19.49 (SD 46.54)	1.89 (SD 46.54)	0.80 (SD 46.54)	7814.27 (SD 8514.47)	<30%: 29.73% 30-50%: 29.73% 50-70%: 13.51% >70%: 29.73%	70.27%	Excellent: 76.92% Very good: 19.23% Good: 3.85%
Deltaswab ViCUM	8	39.93 (SD 20.46)	1.89 (SD 0.23)	1.18 (SD 0.37)	7467.76 (SD 13984.33)	<30%: 25.00% 30-50%: 25.00% 50-70%: 12.50% >70%: 37.50%	62.50%	Excellent: 80.00% Very Good: 20.00%
Deltaswab Virus	54	11.44 (SD 19.53)	1.87 (SD 0.10)	0.47 (SD 0.58)	7814.27 (SD 10181.38)	<30%: 38.89% 30-50%: 14.81% 50-70%: 3.70% >70%: 42.59%	66.67%	Excellent: 83.33% Very good: 11.11% Good: 5.56%
Sigma Virocult	20	24.80 (SD 25.71)	1.86 (SD 0.03)	0.68 (SD 0.34)	19425.12 (SD 12966.48)	<30%: 35.00% 30-50%: 30.00% 50-70%: 5.00% >70%: 10.00%	70.00%	Excellent: 78.57% Very Good: 14.29% Good: 7.14%
Vircell	6	10.53 (SD 5.27)	1.86 (SD 0.06)	0.44 (SD 0.17)	5810.98 (SD 3414.83)	<30%: 83.33% >70%: 50.00%	50.00%	Excellent: 66.67% Very Good: 0.00% Good: 33.33%
None (bronchoaspirates)	6	56.66 (SD 150.03)	1.81 (SD 0.12)	0.91 (SD 0.63)	11885.32 (SD 12894.06)	30-50%: 16.67% 50-70%: 33.33% >70%: 50.00%	83.33%	Excellent: 100%

All of the studied viral transport media comply with the European Union’s (EU mandatory conformity certification and are authorized for use in clinical laboratories. A260/280 and A260/230: Absorbance ratios measured with NanoDrop 2000. DV200: Percentage of fragments > 200 nucleotides, measured with the Agilent Bioanalyzer. nCounter performance: Excellent (at least 3 housekeeping genes with more than 100 lectures; Very Good (at least 2 housekeeping genes with more than 100 lectures and at least 1 housekeeping gene with more than 70 lectures), Good (1 housekeeping gene with more than 100 lectures and at least 2 housekeeping genes with a minimum of 70 lectures). SD: Standard Deviation.

### Gene Expression

After heat inactivation and RNA extraction, RNA eluates were stored at -80°C until further processing. Samples were prepared following manufacturer’s instructions and diluted, in the case of the most concentrated samples, so that the final volume of 5 μl per well contained 150-200 ng of total RNA. Samples were hybridized 22 hours at +65°C and stored at +4°C until digital readout. Results were obtained with the nCounter Prep Station and Digital Analyzer set at high sensitivity. Performance of the NanoString platform and panel validity was assessed with respect to positive and negative controls as well as to the number of readings of housekeeping genes ( Table 2).

### Data analysis

The differential expression analysis data model preferentially applies the optimal statistical method per gene given the following variable distribution: 1) Mixture negative binomial model, 2) Simplified negative binomial model, 3) Log-linear model, in that order. FDR p-value adjustment will be performed according to the Benjamini-Hochberg method. All results are normalized using the geometric mean of the housekeeping genes.

### Limitations of the study

Our study had several strengths, as being the first study in to set up a protocol for the digital multiplexed gene expression analysis of nasopharyngeal exudates using the NanoString nCounter System. On the other hand, and apart from the lack of previous studies on this topic, our study also had other limitations: Since our study is based on the analysis of diagnostic surpluses, we have only been able to analyze a certain number of samples preserved in each viral transport medium. We neither know which of the original nasopharyngeal samples were stored at -80°C and subsequently thawed before performing the diagnostic PCR during the care process, which could explain why RNA in some samples was quite degraded. Finally, reagents shortage during the first and second waves of the COVID-19 pandemic, has also conditioned the techniques used.

## Conclusions

COVID-19 is a major global health problem, making it necessary to develop tools to optimize healthcare and facilitate personalized treatment. From a clinical perspective, the identification of gene transcripts related to the poor prognosis of patients hospitalized with SARS-CoV-2 has undeniable practical value. This complementary information would be straightforward to design multiplexed panels and prediction tools that can be incorporated into computers used in daily practice, helping clinicians predict and identify possible outcomes and facilitating decision-making in this respect.

Our study may also provide useful information to help establish the protocols of other studies based on RNA analysis from nasopharyngeal swab samples using the NanoString nCOUNTER platform.

## Data availability

Normalization, differential expression analysis and pathway analysis can be performed with Nanostring nCounter nSolver™ 4.0 (RRID:SCR_003420), using the Nanostring Advanced Analysis Module 2.0 plugin and following the Nanostring Gene Expression Data Analysis Guidelines. Advanced Analysis Module 2.0 plugin https://www.nanostring.com/products/analysis-solutions/ncounter-advanced-analysis-software/ and following the NanoString Gene Expression Data Analysis Guideliness. Advanced Analysis Module 2.0 software uses open-source R programs for quality control, normalization, differential data analysis, pathway scoring and gene-set enrichment analysis.

## Author contributions

MGA, SGR, IBM, MR conceptualization of the study. SGR, MGA, MR funding acquisition. MGA, ILR, TD, VDL, IBM, MA, MLH investigation and methodology. MPR, IBM, MGA, MA contributed to data analysis. MGA, IBM, MR supervised the study and revised the manuscript. All authors read and approved the final manuscript.

## Ethics

Our Institutional Review Board (CEI Costa del Sol exp.003_JUL20_PI-IMMU-COVID19) approved this study in July 2020. All patients were informed of the study and invited to participate. All participation was subject to the provision of informed written consent.
