# Supplementary material for: Laboratory protocol for the digital multiplexed gene expression analysis of nasopharyngeal swab samples using the NanoString nCounter system
Source: F1000Res. 2022 Sep 30;11:133. Originally published 2022 Feb 2. [Version 2] doi: 10.12688/f1000research.103533.2 (PMC9617068; doi:10.12688/f1000research.103533.2)
Supplement: Peer review history

# Peer review: Reviewer response for version 2

**DOI:** 10.5256/f1000research.138941.r152138

**Published:** 2022-10-28

**Authors:** Tara K. Sigdel

**Affiliations:** 1 Division of MultiOrgan Transplantation, Department of Surgery, University of California San Francisco, San Francisco, CA, USA

The authors have adequately responded to my comments/concerns. I do not have further comments.

Is the rationale for developing the new method (or application) clearly explained?

Yes

Is the description of the method technically sound?

Yes

Are the conclusions about the method and its performance adequately supported by the findings presented in the article?

Partly

If any results are presented, are all the source data underlying the results available to ensure full reproducibility?

Yes

Are sufficient details provided to allow replication of the method development and its use by others?

Yes

Reviewer Expertise:

Organ transplantation, kidney, Biomarker, Transcriptomics, Proteomics

I confirm that I have read this submission and believe that I have an appropriate level of expertise to confirm that it is of an acceptable scientific standard.

# Peer review: Reviewer response for version 2

**DOI:** 10.5256/f1000research.138941.r152139

**Published:** 2022-10-21

**Authors:** Jan Weber

**Affiliations:** 1 IOCB Gilead Research Center, Institute of Organic Chemistry and Biochemistry of the Czech Academy of Sciences, Prague, Czech Republic

Authors have satisfactorily addressed most of my concerns and explained why they used less than optimal approach. They included a paragraph with limitations that explains the large variations in RNA quantity and quality. It is clear that more samples and prepared in more controlled approach are necessary before we can conclude that nasopharyngeal swabs can be used for NanoString nCounter Gene Expression Assay.

 Minor issues:

1. In Table 2 Copan UTM-RT row, put correct SD for A260/280 and A260/280 ratios.

Is the rationale for developing the new method (or application) clearly explained?

Yes

Is the description of the method technically sound?

Partly

Are the conclusions about the method and its performance adequately supported by the findings presented in the article?

No

If any results are presented, are all the source data underlying the results available to ensure full reproducibility?

Partly

Are sufficient details provided to allow replication of the method development and its use by others?

Partly

Reviewer Expertise:

Virology and molecular biology

I confirm that I have read this submission and believe that I have an appropriate level of expertise to confirm that it is of an acceptable scientific standard.

# Peer review: Reviewer response for version 1

**DOI:** 10.5256/f1000research.113351.r122366

**Published:** 2022-03-22

**Authors:** Tara K. Sigdel

**Affiliations:** 1 Division of MultiOrgan Transplantation, Department of Surgery, University of California San Francisco, San Francisco, CA, USA

The method on the digital multiplexed gene expression analysis of nasopharyngeal swab samples using the NanoString nCounter system is adequately presented in the manuscript. This could be of interest to the readers of F1000Research and researchers working with gene expression of immune signature and SARS-CoV-2. 

 I find the rationale of the protocol adequate. I find the protocol not quite complete. Such as: 

1. The authors should provide information on any failure in extracting enough total RNA as demanded by the method.
2. In real-life situations it is hard to get enough RNA from 100% samples. If it was the case then it has to be stated clearly.
3. There is some ambiguity on the amount of total RNA. From the manuscript it appears that only 100 ng is needed. The following statement is contradicting: 
*"4. Add 5 μl of the samples to each corresponding well (RNA concentration should be 100 μg/μl. Dilute out-of-range samples with RNAse-free water)." *

Is the rationale for developing the new method (or application) clearly explained?

Yes

Is the description of the method technically sound?

Yes

Are the conclusions about the method and its performance adequately supported by the findings presented in the article?

Partly

If any results are presented, are all the source data underlying the results available to ensure full reproducibility?

Yes

Are sufficient details provided to allow replication of the method development and its use by others?

Yes

Reviewer Expertise:

Organ transplantation, kidney

I confirm that I have read this submission and believe that I have an appropriate level of expertise to confirm that it is of an acceptable scientific standard, however I have significant reservations, as outlined above.

# Response

**Published:** 2022-03-29

**Authors:** Marilina García Aranda

**Affiliations:** Hospital Costa del Sol, Spain

We thank the reviewer for the constructive comments which will help us to improve the quality of our work. We hope that the revisions in the manuscript and our accompanying responses will be sufficient to make our manuscript suitable for acceptance.

 Please find below our point-to-point responses to the comments.

 The method on the digital multiplexed gene expression analysis of nasopharyngeal swab samples using the NanoString nCounter system is adequately presented in the manuscript. This could be of interest to the readers of F1000Research and researchers working with gene expression of immune signature and SARS-CoV-2. 

 I find the rationale of the protocol adequate. I find the protocol not quite complete. Such as: 

1. The authors should provide information on any failure in extracting enough total RNA as demanded by the method. In real-life situations it is hard to get enough RNA from 100% samples. If it was the case then it has to be stated clearly.
*We agree with the Reviewer and will include the success rate of the extractions performed with the MagCore robot:* *The success rate of extractions performed with the MagCore robot has been 86%.*
2. There is some ambiguity on the amount of total RNA. From the manuscript it appears that only 100 ng is needed. The following statement is contradicting: 
  "4. Add 5 μl of the samples to each corresponding well (RNA concentration should be 100 μg/μl. Dilute out-of-range samples with RNAse-free water)." 
*We agree with the reviewer and apologize for error. We will modify the corresponding paragraphs and will also include additional information:*
*NanoString input recommendations stipulate a total RNA amount range of 100-300 ng and specific ratios of absorbance at 260nm and 280nm (A260/280) measured with NanoDrop within the range 1.8-2nm.* *Given the characteristics of the samples, we decided to hybridize the nCounter probes with 100 ng to 200 ng of total RNA per assay, reason why we only* *assessed gene expression on eluates with total RNA concentration > 2.5 ng/µl measured with the bioanalyzer.*
*Samples were prepared following manufacturer’s instructions. When necessary, we diluted samples in order to obtain 100-200ng of total RNA to each corresponding well. Time of hybridization was set between 16 and 21 hours. Results were obtained with the nCounter Prep Station and Digital Analyzer set at high sensitivity.*

# Peer review: Reviewer response for version 1

**DOI:** 10.5256/f1000research.113351.r122369

**Published:** 2022-02-21

**Authors:** Jan Weber

**Affiliations:** 1 IOCB Gilead Research Center, Institute of Organic Chemistry and Biochemistry of the Czech Academy of Sciences, Prague, Czech Republic

Authors here described their protocol for RNA preparation from nasopharyngeal swabs for subsequent analysis using the NanoString nCounter Gene Expression Assay. This article is a combination of method development with a description of a study on 250 SARS-CoV-2 positive patients with severe COVID-19.

**Major issues**

1. The major criticism here is that readers will not be able to assess if the RNA preparation from nasopharyngeal swab and subsequent evaluation by NanoString platform delivered good and reliable data. Reviewer assumes that it will be part of future publication, but without this data it is difficult to judge that nasopharyngeal swabs can be used as source material for this platform. Ideally, authors can post a preprint into archives and put link here.
2. Authors compared manual RNEasy kit that purifies total RNA (usually from cells, tissue, etc.) with automated kits specialized for purification of viral nucleic acid. Authors should have used e.g. QIAamp MinElute Virus kit or similar for a fair comparison.
3. Authors mentioned the nCounter Human immunology V2 CSO panel that can analyze 594 host genes. For this panel, total RNA isolation kit would be a better choice.
4. Did authors test the platform also with the RNAs purified from EZ1 Virus Mini kit? The A260/A230 ratio is not very good, but high A260/A280 does not necessarily indicate contaminants. Did authors get chance to check the whole UV range spectrum?
5. It would be beneficial to show more data about different viral transport medium, heat inactivation, different nucleic acid extraction protocol and compare their impact on results from several internal controls, rather than showing manufacturer’s instruction for NanoString assay. Was there really any change in the protocol in the case of RNA from nasopharyngeal swabs? If yes, please stress the differences only.

**Minor issues:**

1. Authors should change the future tense to past tense when describing their study in paragraphs “Patients”, “Data analysis” and “Ethics”.
2. Table 1 correct ARN to RNA.
3. Include number of samples “n” per each isolation method in the Table 1.
4. Make clear protocol point no. 4. At the protocol beginning, it is mentioned using 100ng of total RNA, but no.4 talks about using 5µl of sample and RNA concentration 100µg/µl.
5. In the protocol include temperature and other details (such as conditions of each spin).

Is the rationale for developing the new method (or application) clearly explained?

Yes

Is the description of the method technically sound?

Partly

Are the conclusions about the method and its performance adequately supported by the findings presented in the article?

No

If any results are presented, are all the source data underlying the results available to ensure full reproducibility?

Partly

Are sufficient details provided to allow replication of the method development and its use by others?

Partly

Reviewer Expertise:

Virology and molecular biology

I confirm that I have read this submission and believe that I have an appropriate level of expertise to state that I do not consider it to be of an acceptable scientific standard, for reasons outlined above.

# Response

**Published:** 2022-03-29

**Authors:** Marilina García Aranda

**Affiliations:** Hospital Costa del Sol, Spain

*We thank the reviewer for taking the time and effort necessary to review the manuscript and sincerely appreciate all valuable comments and suggestions which will help us to improve the quality of the paper. Please find below* *our point-by-point responses to each of the comments:*

 Authors here described their protocol for RNA preparation from nasopharyngeal swabs for subsequent analysis using the NanoString nCounter Gene Expression Assay. This article is a combination of method development with a description of a study on 250 SARS-CoV-2 positive patients with severe COVID-19.

Major issues

1. The major criticism here is that readers will not be able to assess if the RNA preparation from nasopharyngeal swab and subsequent evaluation by NanoString platform delivered good and reliable data. Reviewer assumes that it will be part of future publication, but without this data it is difficult to judge that nasopharyngeal swabs can be used as source material for this platform. Ideally, authors can post a preprint into archives and put link here.
*We agree with the reviewer. However, we would like to point out that, as a pre-protocol study, we only have preliminary results. We are currently generating additional data and we will be able to stablish more consolidated conclusions in the protocol paper.*
2. Authors compared manual RNEasy kit that purifies total RNA (usually from cells, tissue, 0etc.) with automated kits specialized for purification of viral nucleic acid. Authors should have used e.g. QIAamp MinElute Virus kit or similar for a fair comparison.
*We agree with the reviewer, however, due to shortage of RNA extraction kits during the COVID-19 pandemic, we were not able to use the QIAmp MinElute Virus kit. Instead, we decided to use RNeasy Mini kit based on previously published papers reporting its use as a valid alternative strategy for viral detection in sputum (*DOI: 10.1016/s0166-0934(01)00284-1) *or nasopharyngeal samples (* https://www.biorxiv.org/content/10.1101/2020.03.20.001008v1.full.pdf), *Besides, the RNeasy Mini Kit is one of the most common commercial kits and the Gold Standard for RNA extraction* *. We will modify the corresponding paragraphs in the text accordingly.*
3. Authors mentioned the nCounter Human immunology V2 CSO panel that can analyze 594 host genes. For this panel, total RNA isolation kit would be a better choice.
*We agree with the reviewer. Indeed, we used RNEasy kit that purifies total RNA. We will modify the corresponding paragraph to make this point clearer.*
4. Did authors test the platform also with the RNAs purified from EZ1 Virus Mini kit? The A260/A230 ratio is not very good, but high A260/A280 does not necessarily indicate contaminants. Did authors get chance to check the whole UV range spectrum?
*We are sorry we did not. Despite its affordability, rapidity and ease of use, the NanoDrop usually does not give perfect and reliable readings, especially in samples with contaminants. For this reason, we measured the A260/280 ratios to get a general idea about the best RNA extraction method within the equipment available in the molecular biology laboratory of our hospital. Once we chose the method that best suited our criteria of 260/280 ratios (1.80-2.0 nm), we assessed the quality and quantity of RNA eluates with the Agilent 2100 Bioanalyzer. We will include a new table with the corresponding results in the manuscript. * 
5. It would be beneficial to show more data about different viral transport medium, heat inactivation, different nucleic acid extraction protocol and compare their impact on results from several internal controls, rather than showing manufacturer’s instruction for NanoString assay. Was there really any change in the protocol in the case of RNA from nasopharyngeal swabs? If yes, please stress the differences only.
*Following the recommendations of the Reviewer, we have added information regarding the impact of transport medium and the heat inactivation protocol. While the former did not influence the experiment performance, the heat inactivation protocol was selected based on the reported standards (DOI: 10.1099/jgv.0.001539). Regarding our in house modifications of the manufacturers protocol to adapt it to this specific type of sample, as the reviewer emphasizes, it is important to make them clear in the manuscript. Therefore, we have modified it including and highlighting the optimized processes. This is of high relevance given that RNA from remnant nasopharyngeal exudates is present at very low concentrations and is highly degraded; indeed the RNA integrity number was less than 6 and the DV200 was less than 30% in most of the samples, what would make them inadequate for RNAseq. These samples are also highly heterogeneous, present great variability from patient to patient, and have been transported with several types of transportation medium. Also sampling procedure varies from center to center. Moreover, the fact that only diagnostic remnants were used implied that only limited volumes were available. For these reasons, the optimization of such a method that detects enough number of genes with this type of diagnostic residual samples is of high importance for performing research in the field.*

Minor issues:

1. Authors should change the future tense to past tense when describing their study in paragraphs “Patients”, “Data analysis” and “Ethics”.
*The original version of the manuscript corresponds to the development phase of the technique, which was carried out prior to patient recruitment, reason why “Patients” and “Ethics” sections were written in future tense. We will modify the corresponding paragraphs accordingly.*
2. Table 1 correct ARN to RNA.
*We apologize for the mistake and will correct the typo in the new version of the manuscript.*
3. Include number of samples “n” per each isolation method in the Table 1.
*We appreciate the Reviewer’s suggestion, and will include the corresponding information in the new version.* 
4. Make clear protocol point no. 4. At the protocol beginning, it is mentioned using 100ng of total RNA, but no.4 talks about using 5µl of sample and RNA concentration 100µg/µl.
*We apologize for the mistake and will change the corresponding paragraph.*
5. In the protocol include temperature and other details (such as conditions of each spin).
*We agree with the Reviewer’s suggestion and will modify the protocol accordingly.*
